# Supraspinatus muscle atrophy in relation to aging with or without shoulder pathology: A radiographic study

**DOI:** 10.1016/j.jcot.2023.102171

**Published:** 2023-05-27

**Authors:** Abdul-Rahman Gomaa, Abdul Ahad, Aziz Haque, Jan Muhammad, Radhakant Pandey, Harvider Pal Singh

**Affiliations:** a Trauma & Orthopaedics Unit, University Hospitals of Leicester NHS Trust, Leicester, UK; b Human Anatomy and Resource Centre, University of Liverpool, Liverpool, UK

**Keywords:** Supraspinatus, Supraspinatus atrophy, Aging, Rotator cuff, Shoulder disease

## Abstract

**Introduction:**

Supraspinatus muscle atrophy is commonly associated with shoulder disease, but the effect of ageing on atrophy is not well understood. It was the aim of this study to investigate this effect using MRI scans in older patients.

**Methods and materials:**

A retrospective review of MRI scans in patients aged >70 years was performed between Jan 2016–Dec 2018.Both normal and abnormal scans were included in the analysis which included quantifying muscle atrophy of the supraspinatus using Thomazeu's occupation ratio.

**Results:**

There were 39 normal shoulder MRI scans with a mean age of 75 years (range: 70–88) and 163 abnormal scans with a mean age of 77 years (range: 70–93). The mean supraspinatus occupation ratio for normal MRI scans was 0.57 (range: 0.33–0.86) and abnormal scans 0.35 (range: 0.17–0.90). Occupation ratio was maintained with advancing until the age of 85 years before undergoing a significant declin following this.

**Conclusion:**

This study has shown that the occupation ratio is significantly reduced with shoulder disease, but normal shoulders do not undergo significant atrophy of supraspinatus tendon with increasing age. An occupation ratio of <0.32 is unlikely to occur in normal shoulders and this awareness may be useful when planning shoulder surgery, specifically shoulder arthroplasty.

## 1 Introduction

Supraspinatus muscle atrophy seen on magnetic resonance imaging (MRI) scans commonly occurs due to shoulder pathologies such as rotator cuff tears and glenohumeral arthritis. It can also occur following nerve injury or disuse over time.^1^ Rotator cuff tears involving the supraspinatus tendon can induce atrophy through chronic unloading, apoptosis and fatty infiltration of the muscle.^1^^,^^2^

Goutallier et al.^1^ and Thomazeau et al.^3^ have described two contrasting ways of grading muscle degeneration in the presence of rotator cuff tears. Goutallier et al.^1^ originally based their grading on CT scans, but it has more recently been applied to MRI scans.^4^ It is a grading system based on the proportion of muscle that has been infiltrated by fatty tissue (whereby grade 0: normal muscle, grade 1: some fatty streaks, grade 2: less than 50% fatty muscle atrophy, grade 3: 50% fatty muscle atrophy, grade 4: greater than 50% fatty muscle atrophy).^1^

Thomazeau et al. have instead described muscle atrophy using MRI scans to calculate the occupation ratio.^3^ This ratio is calculated by measuring the cross-sectional area of the supraspinatus muscle belly and comparing it to its fossa in the scapular Y view. The occupation ratio is then classified into three grades with higher grades suggesting greater loss of muscle bulk (Grade 1: ≥0.60, Grade 2: 0.40–0.59.9, Grade 3: <0.40).^3^ Both of these grading systems correlate with histological changes occurring within the muscle following cuff tears as well as other radiological findings such as tear size and tendon retraction.^5^

Rotator cuff atrophy seen on MRI scans in the absence of shoulder disease in the elderly may significantly impair activities of daily living and also have an impact on decision making choice between anatomic and reverse total shoulder arthroplasty. The effect of ageing on supraspinatus muscle atrophy is poorly understood.^6^ Furthermore, animal studies have shown that age is likely to affect rotator cuff muscle function adversely.^7^

This study aims to evaluate, through radiographic means, the degree of supraspinatus muscle atrophy in relation to age, both in the presence and absence of shoulder pathology, using MRI scans.

## 2 Material and methods

A retrospective review of all shoulder MRI scans performed within our institution between January 2016 and December 2018 was conducted following institutional review board approval (reference number: 10310).

Inclusion criteria were: patients above the age of 70 years, MRI scans of the shoulder with a validated radiology report and availability of the lateral most sagittal section, including the scapula spine and coronal view of the suprascapular notch.

Initial evaluation of all MRI scans was performed by two experienced reviewers (senior orthopaedic consultants) to classify the normality and quality of rotator cuff muscles using Thomazeau's grading system and the Goutallier stage.^1^^,^^3^ These scans were further reviewed by the senior author (HPS) of the paper.

Whilst the original Goutallier staging was based on CT scans, more recently this has been validated for use with MRI scans and as such, this technique was employed for the included MRI scans.^1^ The included MRI scans were non-contrast studies performed for investigating potential causes of shoulder pain, weakness, stiffness, radicular pain, symptoms of instability as well as neoplastic lesions. The MRI scans were requested either by general practitioners in the community or by specialists in secondary care, including orthopaedic surgeons and other musculoskeletal specialists such as physiotherapists and rheumatologists. The most common indication of the scan was ‘shoulder pain’. An MRI scan was deemed normal if it showed no abnormalities upon review by both the reporting radiologist and the orthopaedic surgeon reviewing it, and if the primary diagnosis was not related to the shoulder joint.

**Fig. 1:**
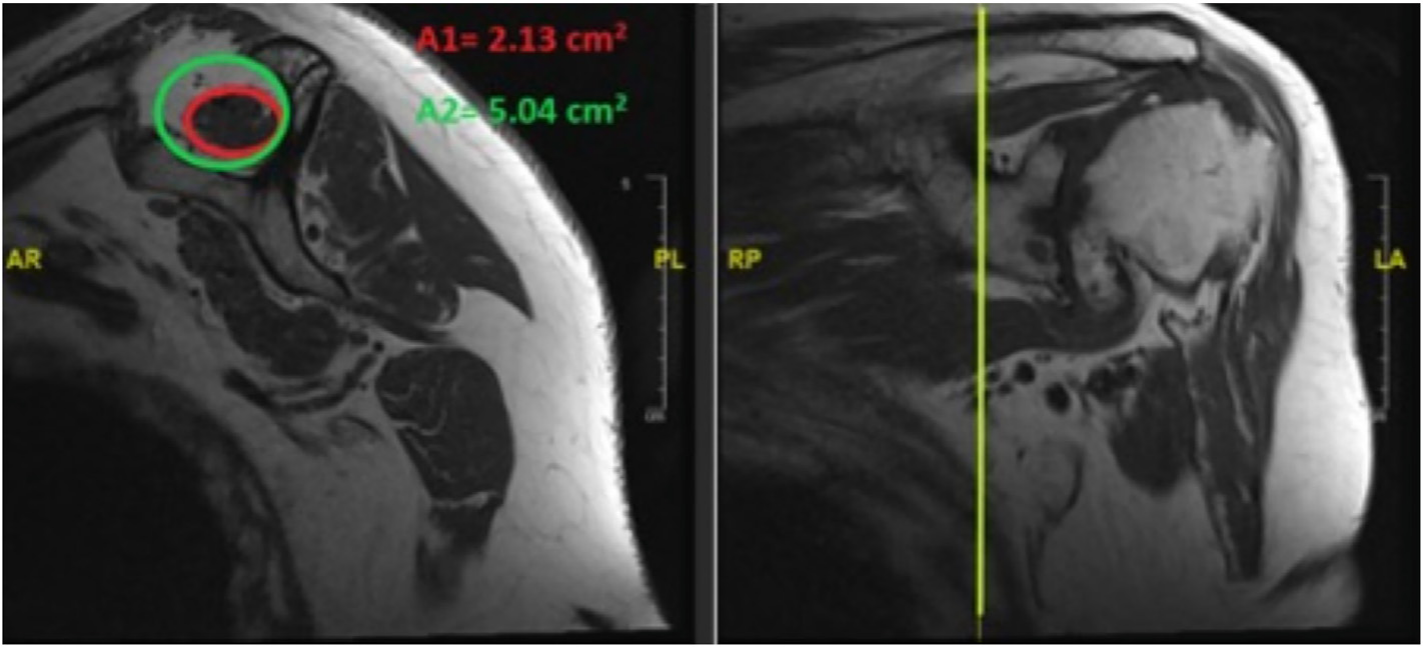
Calculation of occupation ratio using the cross-sectional area of the supraspinatus muscle and its fossa on a sagittal MRI image.

Using the included shoulder MRI scans, the cross-sectional area of the supraspinatus muscle and its fossa was calculated on a sagittal image called the “Y" shaped view.^3^ This view is the scapula's lateral most image in the sagittal plane showing the scapular spine posteriorly, the coracoid process anteriorly and the scapula body inferiorly. Using this as a reference point the occupation ratio was then calculated by using the method described by Khoury et al.,^8^ in which cross section area of the muscle and fossa estimated by drawing the best circle or ellipse by using the “ellipse” tool provided by PACS manufacturer. (Picture Archiving and Communications systems version 2 GE Monogram, United Kingdom). These ellipses or circles need to be sited around the supraspinatus belly and the bony fossa on the “Y” view. At this cross section the supraspinatus fossa is mostly limited by bone. An ellipse is drawn to approximate the supraspinatus muscle belly and its fossa as shown in Fig. 1.

The occupation ratio was then calculated using the cross-sectional area of the two approximations described by Thomazeau et al.^3^ This ratio was calculated by dividing cross section area or supraspinatus tendon to the suprascapular fossa. The ratio is graded as grade 1 (≥0.60 occupation ratio of the muscle belly within the fossa), grade 2 (0.40–0.59.9) and grade 3 (<0.40).^3^ Independent t-tests were performed for normally distributed data. The relationship between age and occupation ratio was tested using linear regression analysis. A P value < 0.05 was deemed statistically significant. Statistical analysis was performed using Microsoft Excel version 16.32.

## 3 Results

A total of 222 shoulder MRI scans were performed for patients aged 70 years and over between January 2016 and December 2018. 20 MRI scans were excluded due to motion artefact or poor Y view delineation on the required images. Table 1 summarises the patient demographics and characteristics of the included 202 patients.

**Table 1:** A summary of the demographics and characteristics of the included 202 patients.

Total	All	Normal	Abnormal
	202	39	163
**Age**			
Mean	76.5	74.9	76.8
s.d.	4.8	4.8	4.8
Mode	70.0	70.0	74.0
Median	76.0	73.0	76.0
Min	70.0	70.0	70.0
Max	93.0	88.0	93.0
**Gender**			
Male	78	16	62
Female	124	23	101
**Occupation ratio**			
Mean	0.3964	0.5737	0.3539
s.d.	0.2170	0.1401	0.2108
Median	0.3858	0.5751	0.3183
Min	0.0170	0.3290	0.0170
Max	0.8965	0.8559	0.8965
**Thomazeau Grade**			
Mild	38	16	22
Moderate	60	19	41
Severe	104	4	100
**Goutallier Stage**			
0	14	5	9
1	39	18	21
2	33	16	17
3	26	0	26
4	90	0	90

After analysing all the MRIs and associated records, it was determined that 39 shoulder MRI scans (19.3%) were categorised as “normal scans,” while the remaining 163 scans (80.7%) showed evident shoulder pathology, predominantly rotator cuff issues, and were classified as the “abnormal scans” group.

The ‘normal scans’ group (n = 39) demonstrated a mean occupation ratio of 0.57 (range: 0.33–0.86; s.d: 0.14). Of them, 23 (59%)were female and 16 (41%) male. Evaluation of MRI scans by gender showed a mean occupation ratio for females to be 0.53 compared to 0.64 for males. Five patients (12.8%) with normal scans had Goutallier stage 0, eighteen patients (46.2%) had Goutallier stage 1 and sixteen patients (41.0%) had Goutallier stage 2.

**Fig. 2:**
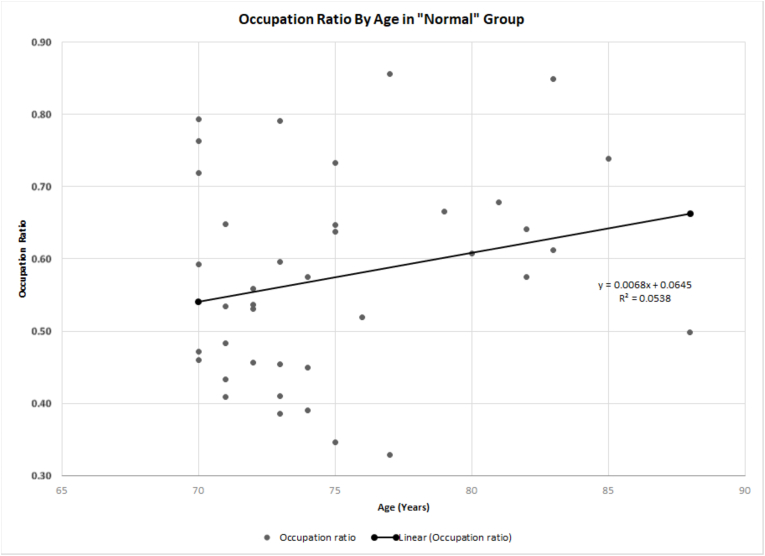
Linear regression between age and occupation ratio in ‘normal scans' group.

**Fig. 3:**
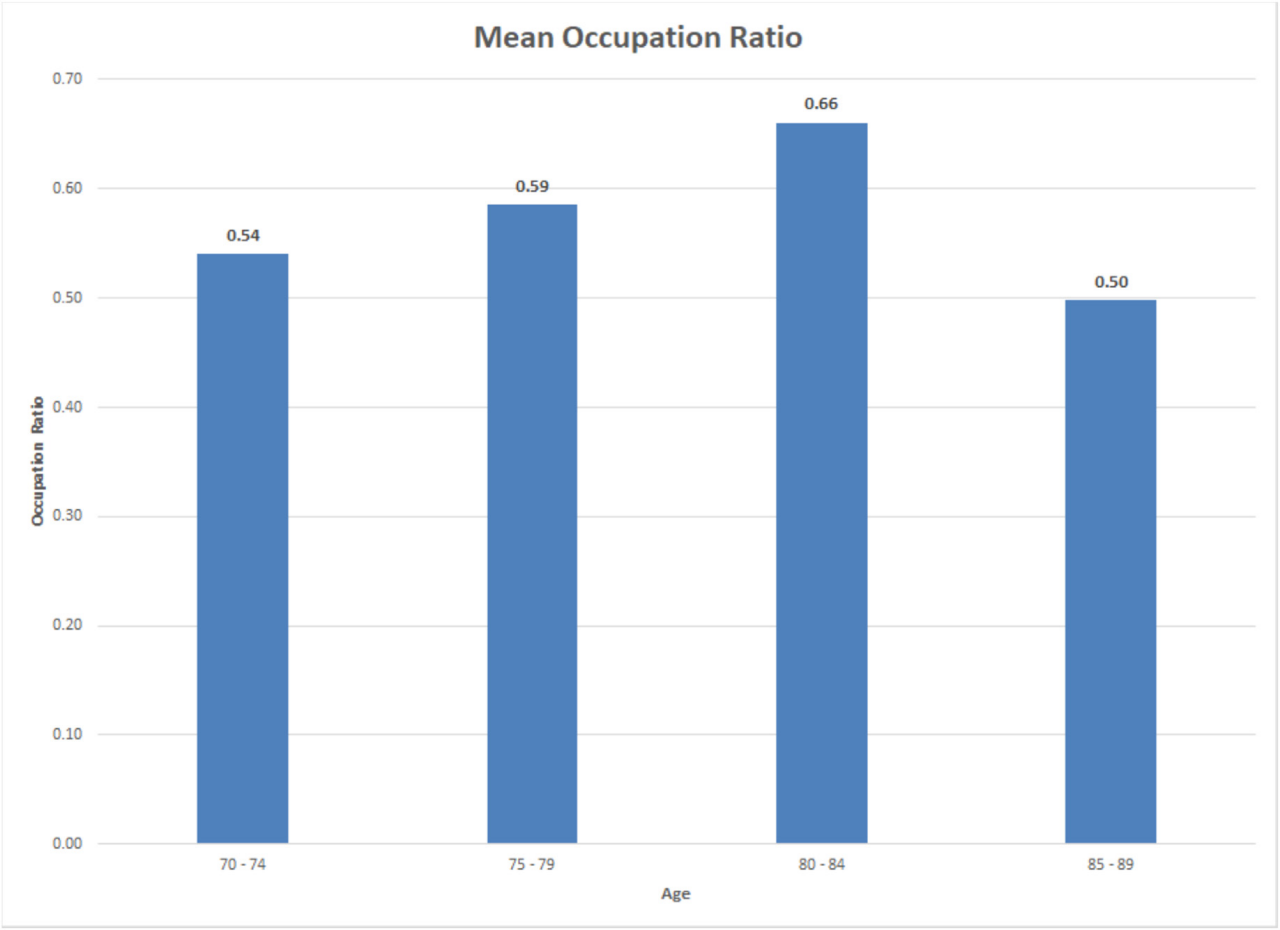
Mean occupation ratio broken down by age range.

Linear regression analysis between age and occupation ratio suggested a weakly positive, non-significant correlation between occupation ratio and advancing age (p = 0.155, Coefficient of 0.0068, R^2^ = 0.0538) (Fig. 2). Conversely, we found a significant difference in occupation ratio between males and female patients (p = 0.015). Further performing simple linear regression analysis on occupation ratio by age for each gender showed significantly different coefficients; whereby the male coefficient was 0.0085 compared to the female coefficient of 0.0052. Furthermore, it was observed that the occupation ratio of the supraspinatus did not exhibit a significant decline between the ages of 70 and 84 years. However, there was a significant decline in this ratio beyond the age of 85. It's worth noting that the occupation ratio never dropped below 0.4 (Fig. 3).

The occupation mean ratio of the ‘normal scans’ group was significantly greater than the ‘abnormal scans’ (p < 0.001). Furthermore, ‘normal scans’ group had a significantly greater proportion of mild and moderate scans according to the Thomazeau grade system (i.e 89.7% compared to 38.7% in the ‘abnormal scans’ group). According to the Goutallier staging system, most (71.2%) of the ‘abnormal scans’ group were between stages 3 and 4; whilst 87.2% of the ‘normal scans’ group were between stages 1 and 2 as outlined in Table 1.

The ‘abnormal scans’ group consisted of 163 (80.7%) shoulder MRI scans showing evidence of pathology. These included: rotator cuff tears of variable thickness and size, glenohumeral joint arthritis, cuff tear arthropathy, lipomatous tumours, severe subacromial impingement and advanced ACJ arthritis. The mean occupation ratio and Goutallier grading of normal and abnormal MRI scan are summarised in Table 2, broken down by the radiographic MRI diagnosis. Among those scans with shoulder pathology 101 (62.0%) were female and 62 (38.0%) were male. The mean occupation ratio for females was 0.34 compared to 0.38 for males.

**Table 2:** Mean occupation ratio and Goutallier Grading of normal and abnormal MRI scans.

	Normal MRI Scans	Full Thickness Tear and Glenohumeral Arthritis	Tendinopathy, Impingement+/-ACJ Arthritis	Glenohumeral Arthritis	Partial Tear	Tendinopathy+/-Partial Tear with Glenohumeral Arthritis	Massive Tear and Glenohumeral Arthritis	Other Pathology
Number of patients	39	42	23	18	13	12	3	52
Mean occupation ratio (Range)	0.57 (0.33–0.86)	0.24 (0.06–0.72)	0.53 (0.28–0.88)	0.41 (0.17–0.89)	0.55 (0.26–0.81)	0.40 (0.16–0.69)	0.10 (0.08–0.11)	0.38 (0.05–0.90)
Goutallier Grade 0 (n, %)	8 (20.5%)	- (0%)	7 (30.4%)	1 (5.6%)	1 (7.7%)	- (0%)	- (0%)	- (0%)
Goutallier Grade 1 (n, %)	9 (23.1%)	2 (4.8%)	3 (13.0%)	1 (5.6%)	7 (53.8%)	- (0%)	- (0%)	8 (15.4%)
Goutallier Grade 2 (n, %)	22 (56.4%)	1 (2.3%)	5 (21.7%)	1 (5.6%)	2 (15.4%)	4 (33.3%)	- (0%)	4 (7.7%)
Goutallier Grade 3 (n, %)	- (0%)	7 (16.7%)	4 (17.4%)	7 (38.9%)	2 (15.4%)	1 (8.3%)	- (0%)	5 (9.6%)
Goutallier Grade 4 (n, %)	- (0%)	32 (76.1%)	4 (17.4%)	8 (44.4%)	1 (7.7%)	7 (58.3%)	3 (100%)	35 (67.3%)

Occupation ratio (<0.2) was significantly lower in MRI scans that demonstrated three or more concomitant pathological findings such as: full thickness tear, glenohumeral arthritis and ACJ arthritis (n = 23). The most common Goutallier stage in this group was 4. It is important to mention that glenohumeral arthritis, either alone or in conjunction with other pathologies, was the most frequent observation on the abnormal MRI scans (n = 75).

Massive tears (>5 cm) associated with glenohumeral arthritis and ACJ arthritis impacted severely on the occupation ratio of the supraspinatus. Amongst them, three patients had previous mal-united proximal humeral fracture causing moderate supraspinatus tendon atrophy with the mean occupation ratio of 0.35 (range: 0.09–0.55). Avascular necrosis (AVN) of the humeral head was associated with significant supraspinatus tendon atrophy. All cases of AVN in this cohort were secondary to proximal humeral fractures with a mean occupation ratio of 0.26 (range: 0.20–0.33). All of them had stage 4 atrophy on Goutallier's staging.

As the shoulder disease progressed, the supraspinatus muscle showed greater atrophy. Specifically, partial tears had lower levels of atrophy compared to tendinopathy, impingement, and ACJ arthritis. These conditions, in turn, showed lower levels of atrophy compared to glenohumeral arthritis. On the other hand, full thickness cuff tears resulted in greater atrophy than all of the aforementioned conditions, but not as much as cuff tear arthropathy.

Linear regression analysis between age and occupation ratio of the ‘abnormal scans’ group suggested a weakly negative, non-significant correlation between occupation ratio and advancing age (p = 0.055, Coefficient of −0.0066, R^2^ = 0.0226).

## 4 Discussion

Shoulder pathologies such as rotator cuff tears and glenohumeral arthritis can lead to muscle degeneration and atrophy over time. However, it is unclear if supraspinatus atrophy occurs with advancing age in otherwise radiographically normal shoulders. This study has shown that the occupation ratio is significantly reduced in shoulder disease, but radiologically normal shoulders do not undergo significant atrophy with increasing age. The occupation ratio did not decline significantly from 70 years of age to 84 years with the mean occupation ratio of 0.57.

When considering patients for total shoulder arthroplasty, the decision making in young patients with intact rotator cuff and in those patients with full thickness cuff tears or signs of cuff tear arthropathy is relatively straight forward. The difficulty arises when surgeons are faced with an older patient with isolated glenohumeral arthritis and an intact cuff with some signs of atrophy and degeneration. There is concern that poor rotator cuff function following an anatomic total shoulder arthroplasty may lead to poorer outcomes; and some of these patients may benefit from a reverse total shoulder arthroplasty.^9^^,^^10^

This study highlights that age itself in the absence of other shoulder disease is unlikely to lead to significant atrophy and if significant atrophy is noted (<0.40 occupation ratio) then care must be taken when deciding between anatomic and reverse total shoulder arthroplasty.

Thomazeu et al.^3^ noted that supraspinatus tendinopathy is not associated with significant muscle atrophy. Our findings support this, with a mean occupation ratio of 0.53 for those scans showing tendinopathy alone. Similarly ACJ arthritis on its own did not lead to significant supraspinatus atrophy. Those patients with multiple pathologies on the MRI scan had significantly higher levels of atrophy.

Goutallier et al.^1^ classified rotator cuff atrophy according to fatty infiltration of the musculature with higher stages signifying increasing fatty infiltration. Fuchs et al.^11^ showed irreversible changes to the cuff muscles in an animal model following delayed repair and retracted tendons. Conversely, there is some evidence that muscle atrophy in the context of massive rotator cuff tears may be reversed if operated on within two years of injury.^12^ In addition, Gulotta et al.^13^ have shown that older patients following rotator cuff repair are less likely recover from muscle degeneration following repair.

Advancing age is at times held responsible for the development of rotator cuff atrophy. By using MRI scans to differentiate between normal and abnormal reported shoulder scans we have shown that age on its own is not likely to be an independent variable contributing to cuff atrophy. However; we did see four patients with no evident shoulder disease on MRI with severe atrophy according to Thomazeau's grading. On closer assessment, it was found that these patients had significant frailty and co-morbidities, suggesting that they may not be physiologically loading their shoulder.

If age was an independent risk factor for rotator cuff atrophy, we would anticipate a higher incidence of shoulder pain as age increases due to compromised shoulder function. However, cross-sectional studies examining the prevalence of shoulder pain with advancing age have not supported this assumption.^1^^,^^14^

Our findings are not necessarily in contrast with Raz et al.^15^ who found that the cross-sectional area of the supraspinatus muscle declines throughout adulthood as we have chosen to concentrate on those patients over the age of 70. It may be that the overall cross-sectional area of the rotator cuff does decline with age if compared to much younger patients, but we have shown that in the absence of shoulder disease, there is a minimum value below which atrophy does not progress even with increasing age. Male patients are less likely to develop muscle atrophy and this is yet another consideration when choosing implants for shoulder arthroplasty in those patients with glenohumeral arthritis and an intact rotator cuff.

### 4.1 Limitations

The study has some limitations, such as a small sample size and no correlation with functional outcomes. The evaluation of supraspinatus atrophy relied solely on MRI scans, and the patients' shoulder strength was not assessed. We verified the absence of shoulder disease based on the MRI report, and patients were not clinically evaluated. Additionally, where the indication for performing the MRI was “shoulder pain,” it could potentially have led to muscle atrophy due to disuse.

## 5 Conclusion

This study has shown that the occupation ratio did not decline significantly with age and remained above 0.40 in those patients with normal reported shoulder scans. A larger study looking to compare muscle atrophy with radiologically normal shoulders and functional outcome scores, EMG studies and histological analysis would offer a better understanding of the impact of age on rotator cuff function. This is an important factor to consider when assessing patients up for total shoulder arthroplasty and deciding between implants in older patients with glenohumeral osteoarthritis.

## Ethics statement

Institutional review board approval for this study was obtained from University Hospitals of Leicester NHS Trust (Reference number: 10310).

## Funding

This research did not receive any specific grant from funding agencies in the public, commercial or not-for-profit sectors.

## Declaration of competing interest

The authors declare that they have no known competing financial interests or personal relationships that could have appeared to influence the work reported in this paper.
